# Evolving strategies of intracellular Hypervirulent *Klebsiella pneumoniae* during phage therapy: Reducing host autophagy and inflammation

**DOI:** 10.1080/21505594.2025.2600148

**Published:** 2025-12-04

**Authors:** Huale Chen, Yao Sun, Zeyu Huang, Deyi Zhao, Jingchun Kong, Huanchang Chen, Cui Zhou, Tieli Zhou

**Affiliations:** a Department of Clinical Laboratory, Key Laboratory of Clinical Laboratory Diagnosis and Translational Research of Zhejiang Province, The First Affiliated Hospital of Wenzhou Medical University, Wenzhou, Zhejiang, China; b Department of Clinical Laboratory, The Second Affiliated Hospital and Yuying Children’s Hospital of Wenzhou Medical University, Wenzhou, Zhejiang, China

**Keywords:** Hypervirulent *Klebsiella pneumoniae*, intracellular survival, phage therapy, immune modulation, autophagy

## Abstract

Hypervirulent *Klebsiella pneumoniae* (hvKp) presents challenges in infection management due to antibiotic resistance associated with its intracellular persistence. This study investigates the efficacy of phage therapy against intracellular hvKp using a two-stage murine model. We assessed changes in virulence, host survival, and immune responses through phagocytosis assays, transmission electron microscopy, and Western blotting, complemented by transcriptomic and proteomic analyses. Results indicate that phage therapy reduces mortality and modulates bacterial virulence by downregulating capsule production. Following phage exposure, hvKp adapts by enhancing its oxidative stress resistance. Crucially, these adaptations weaken host inflammatory and autophagy responses, enabling better survival within host cells. These adaptations suggest that while phage therapy can mitigate infection severity, the capacity of hvKp to modulate host pathways underscores the complexity of treating intracellular infections and highlights the importance of targeting both bacterial and cellular responses.

## IMPORTANCE

In this study, we investigated the use of phage therapy against intracellular infections caused by hypervirulent Klebsiella pneumoniae, a bacterium capable of evading antibiotics by residing within host cells. Using a mouse model, we demonstrated that phage therapy improves survival and reduces bacterial virulence. However, exposure to phages prompted the bacterium to enhance its oxidative stress defenses and adapt by suppressing host inflammatory and autophagy responses. These adaptations not only promote bacterial survival within host cells but also result in higher bacterial loads in tissues and organs, potentially leading to chronic infections. This poses a severe threat to immunocompromised individuals, underscoring the dual challenge of phage therapy: while it offers significant therapeutic potential, bacterial adaptations can undermine its efficacy. Our findings provide crucial insights into the host-pathogen dynamics during phage therapy, paving the way for more effective strategies to combat intracellular bacterial infections.

## GRAPHICAL ABSTRACT

**Figure:**
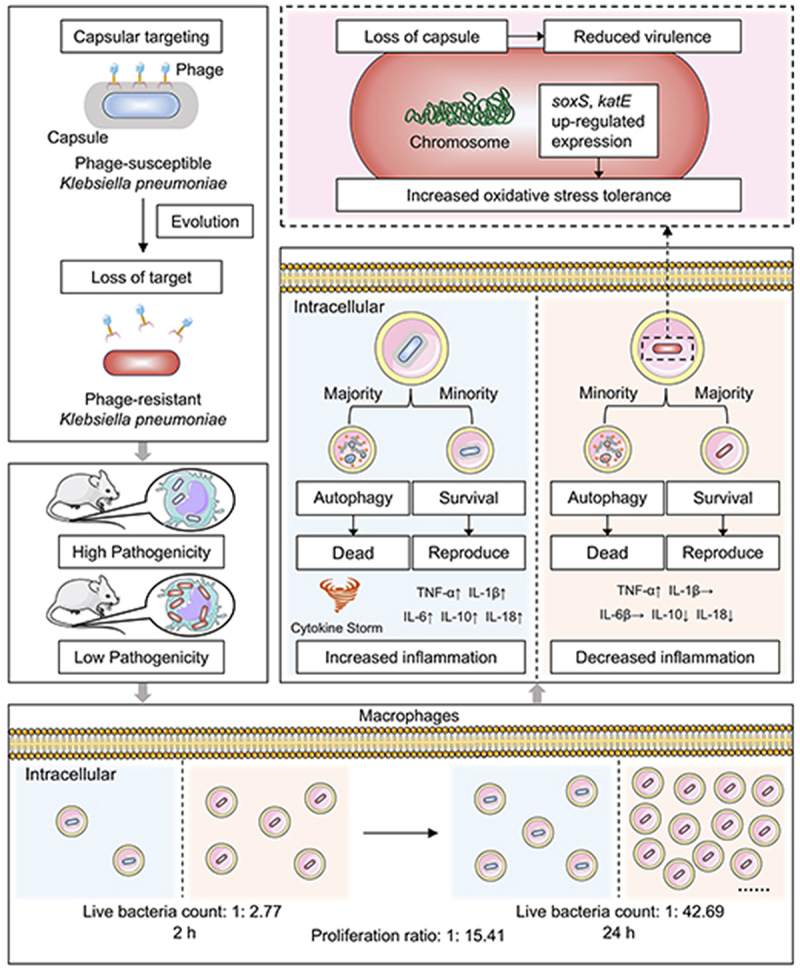

## Introduction

*Klebsiella pneumoniae* (*K. pneumoniae*) has emerged as a substantial global health concern, as identified by the World Health Organization [1,2]. Hypervirulent *K. pneumoniae* (hvKp) is a major cause of severe community-acquired and nosocomial pneumonia, especially in elderly or immunocompromised patients, and is frequently associated with high-mortality bacteremia [3–7]. Meanwhile, recent findings challenge the traditional view of hvKp as purely extracellular, demonstrating its capability to persist intracellularly, disrupt lysosome-vacuole fusion in macrophages, and induce a distinctive macrophage polarization toward an M(Kp) phenotype, which is specific to hvKp infection and influences host immune responses [5,7,8]. Many bacterial pathogens can evade antibiotics that poorly penetrate eukaryotic cell membranes by residing intracellularly, thereby exhibiting tolerance to cell-impermeable drugs such as aminoglycosides [9–13]. By subverting macrophage polarization, intracellular hvKp may exploit these cells as “Trojan horses” for metastatic dissemination, increasing the risk of secondary infections and persistent, hard-to-treat foci that evade both antibiotics and immune clearance [6,8].

Phages, often described as “living antibiotics,” present unique advantages over traditional antibiotics, including their selective targeting of pathogens, preservation of host microbiota, efficacy against drug resistant strains, and minimal toxicity [14–17]. Research indicates that phages, capable of entering mammalian cells and maintaining infectivity, present a promising approach to targeting intracellular bacteria [18]. Although phage therapy has shown effectiveness in early trials, its application to intracellular infections faces challenges due to the emergence of phage-resistant bacteria, which, while occasionally reducing bacterial fitness, have not consistently yielded clinical success, thus posing ongoing challenges for development [6,15,19–21].

In evaluating the efficacy of phage therapy against intracellular infection by hvKp, we established a murine model based on an existing method to simulate intracellular hvKp infection and administered phage treatment [9]. While the therapy significantly reduced mortality, our findings reveal that hvKp can evolve phage resistance within host cells, potentially enabling it to persist and significantly increase bacterial loads in host organs despite therapeutic intervention. This adaptation by intracellular hvKp indicates a potential for more stubborn infections, emphasizing the urgent need to develop effective countermeasures against phage resistance. Our findings emphasize the dynamic interaction between microbial evolution and treatment strategies, calling for continuous advancements in phage therapy research. Here, we specifically examine how phage pressure drives the intracellular adaptation of hvKp, and how such evolutionary trajectories influence macrophage inflammation and autophagy, ultimately shaping host-pathogen dynamics.

## Results

### Decreased mortality yet increased bacterial burden: paradoxical outcomes of phage therapy in intracellular hvKp infections

**Figure 1.**
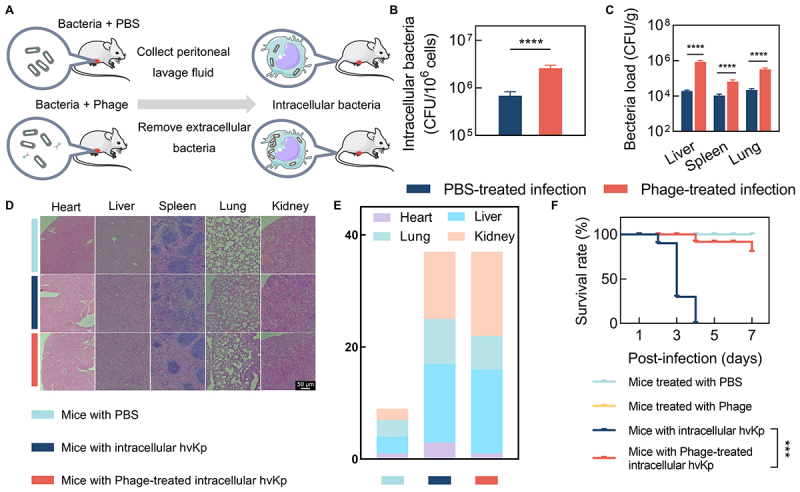
Outcomes of phage therapy on intracellular hvKp infections in a mouse model. (A) illustration of the intracellular bacterial infection model setup; (B) intracellular bacterial counts in host peritoneal macrophages and neutrophils at 24 hours post-infection, each treatment group included 6 mice, data were analyzed by an unpaired t test; (C) bacterial counts in organ tissues at 48 hours post-infection, each treatment group included 6 mice, data were analyzed by an unpaired t test; (D) histological examination (he staining) of organ tissues at 48 hours post-infection; (E) quantitative pathology scores of organs tissues at 48 hours post-infection. F: survival rates of mice with bloodstream infections due to intracellular bacteria, each treatment group included 10 mice, data were analyzed by a mantel-cox log-rank test.

We initially determined that a MOI of 0.1 is the minimum concentration that effectively postpones the emergence of phage resistance to the greatest extent, with no further postponement observed at higher concentration (S1 Fig). In the first stage of our intracellular bacterial infection model in mice, intracellular hvKp was treated with ΦK2044 at a MOI of 0.1. This allowed us to assess the intracellular survival of hvKp in the control group and evaluate the phage’s ability to reduce intracellular bacterial load in the treated group. In the second stage, these differently treated intracellular hvKp were injected into mice via the tail vein to assess their pathogenicity within host peritoneal cells, thereby evaluating the effect of phage therapy on intracellular bacteria (Figure 1(A)). After treating with phages, an unexpected outcome was observed: a 3.78-fold increase in bacterial loads within host peritoneal cells compared to the untreated group, which was infected with bacteria but without phage exposure (Figure 1(B)). Similarly, the bacterial loads in the liver, spleen, and lungs were significantly higher in mice infected with phage-treated intracellular bacteria compared to those in the untreated group, as determined by microtiter plate colony counting (Figure 1(C)). However, no viable bacteria were detected in the heart and kidneys, with levels below the detection limit of 100 CFU/mL for this method. Substantial histopathological changes were evident in the liver, spleen, and lungs of mice 48 hours post-infection (Figure 1(D)). Specifically, the liver showed increased spacing between cells, the spleen exhibited a noticeable decrease in the red to white blood cell ratio, and the lungs displayed non-expansion of alveoli. These changes were observed in both the untreated (infected only with hvKp and treated with PBS) and phage-treated groups. However, no significant pathological changes were detected in the heart and kidneys of any group. Pathology scores – which sum the severity of damage across heart, liver, lung, and kidney (Y-axis shows total score) – were significantly elevated at 37 in both the untreated and phage-treated intracellular hvKp-infected groups, compared to a substantially lower score of 9 in the uninfected control group (Figure 1(E)). Mice infected with untreated intracellular hvKp exhibited 100% mortality within four days, whereas those infected with phage-treated intracellular hvKp showed a significantly reduced mortality rate, with 80% survival after seven days (Figure 1(F)).

**Figure 2.**
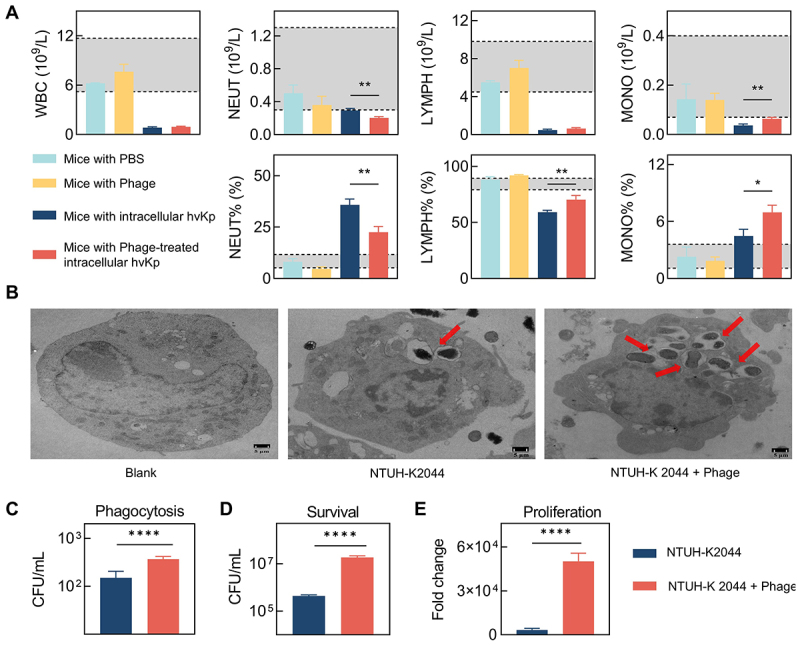
Intracellular survival and dynamics of hvKp within macrophages. (A) hematological analysis of mice 48 hours post-infection with intracellular bacteria (the gray area represents normal ranges), each treatment group included 3 mice, data were analyzed by an unpaired t test; (B) visualization of intracellular bacteria within RAW264.7 macrophages by transmission electron microscopy, 24 hours post-infection; (C) bacterial phagocytose by mouse macrophages RAW264.7, 2 hours post-infection, data were analyzed by an unpaired t test; (D) Count of bacteria surviving within mouse macrophages RAW264.7, 24 hours post-infection, data were analyzed by an unpaired t test; (E) growth ratio of intracellular bacteria in mouse macrophages RAW264.7 calculated as 24-hour survival relative to initial phagocytosis at 2 hours post-infection, data were analyzed by an unpaired t test.

Hematological analysis indicated that phage treatment alone did not significantly affect murine blood cell counts, with all metrics within normal ranges (Figure 2(A), gray area). However, mice with intracellular infections exhibited abnormal blood parameters, with significant reductions in total white blood cells, lymphocytes, and monocytes below normal levels. Notably, among these infected mice, those treated with phages had relatively higher monocyte counts, whereas those untreated with phages exhibited higher neutrophil counts. Transmission electron microscopy of RAW264.7 macrophages exposed to different treatments revealed a greater presence of intracellular bacteria in the phage-treated group (Figure 2(B)). Consistently, colony counting further confirmed that, compared with the infection group treated with PBS, macrophages infected with hvKp from the phage-treated group exhibited significantly higher bacterial uptake at 2 hours post-infection, as well as enhanced intracellular survival and proliferation at 24 hours post-infection (Figure 2(C-E)).

### Reduced virulence of intracellular hvKp post-phage treatment

**Figure 3.**
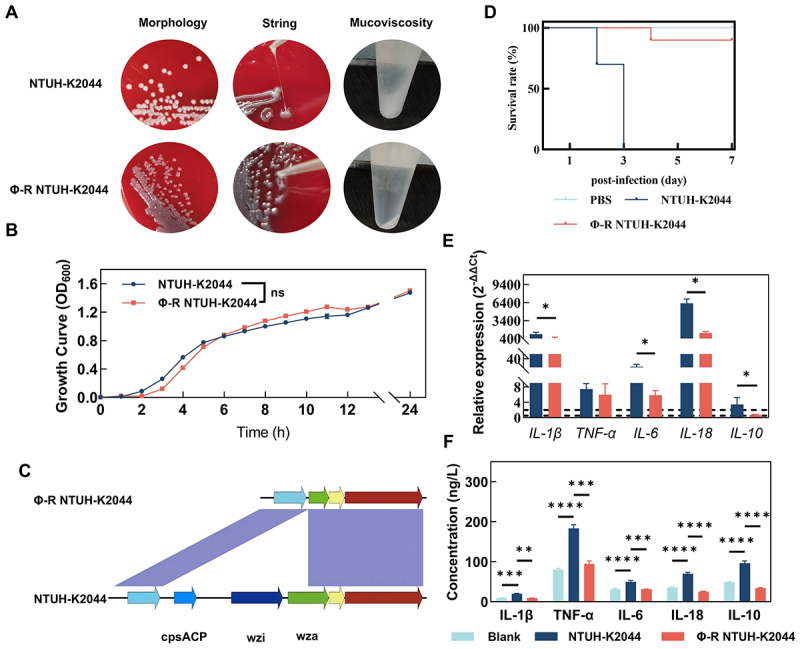
Reduction in virulence of hvKp after phage treatment. (A) colony morphology, filamentation, and capsule images; (B) growth curves; (C) genomic comparison diagram; (D) lethality results from mouse peritoneal infection, each treatment group included 10 mice; (E) gene expression levels of inflammatory factors (qRT-PCR, normalized to baseline in untreated RAW264.7 cells); (F) expression levels of inflammatory factors (ELISA).

Intracellular bacteria from two distinct treatment groups were isolated and propagated for ten generations under non-stress conditions. Significant morphological changes were observed in phage-treated bacteria (Φ-R NTUH-K2044), with colonies turning gray and mature colonies unable to produce filaments, a morphology consistent with capsule loss; centrifugation did not reveal the presence of mucilage (Figure 3(A)). Growth curve analysis revealed no significant differences in the proliferation rates between NTUH-K2044 and Φ-R NTUH-K2044 (Figure 3(B)). Whole-genome sequencing of Φ-R NTUH-K2044 identified a deletion exceeding 4000 bp within the capsule coding region affecting the *wza*, *wzi*, and *cpsACP* genes (Figure 3(C)). *In vivo* assessments via a murine peritoneal infection model demonstrated a significant reduction in lethality from 100% in the control NTUH-K2044 to 10% in the phage-treated strain, designated Φ-R NTUH-K2044 (Figure 3(D)). Quantitative Real-time PCR of inflammatory cytokines indicated elevated levels of *IL-1β*, *TNF-α*, *IL-6*, *IL-18* and *IL-10* upon NTUH-K2044 stimulation. However, *TNF-α* levels showed no significant decrease, and although all other cytokines were significantly less elevated in the Φ-R NTUH-K2044 stimulated group compared to the NTUH-K2044 stimulated group, they were still higher than in the uninfected controls (Figure 3(E)). ELISA assays confirmed these observations, showing that all measured inflammatory markers were significantly elevated after stimulation with NTUH-K2044. In contrast, in the NTUH-K2044 stimulated group, only TNF-α levels were elevated compared to uninfected controls, albeit significantly lower than levels induced by NTUH-K2044 stimulation. The levels of IL-1β, IL-6, IL-18, and IL-10 showed no significant differences from uninfected baseline levels (Figure 3(F)). Consistent with these observations, the composite InflammationScore was strongly and inversely associated with the survival proportion (Spearman ρ = −0.878, twosided *p* = 2.39 × 10^−4^; *n* = 6). Additionally, no significant impact was observed on the overall inflammatory cytokine production in macrophages treated solely with phage (S2 Fig), nor was there a notable influence on the intracellular survival of phage-resistant strains (S3 Fig).

### Enhanced oxidative stress resilience in hvKp following phage therapy: bacterial adaptations for increased intracellular viability

**Figure 4.**
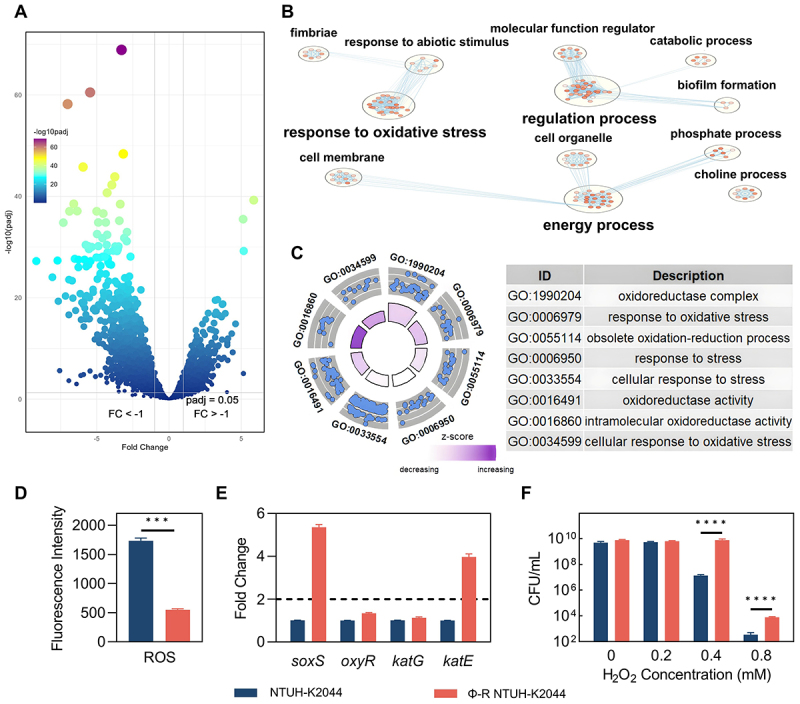
Enhanced oxidative stress resistance in hvKp post-phage treatment. (A) volcano plot of differentially expressed genes in the transcriptome of hvKp before and after phage treatment; (B) go enrichment network diagram of differentially expressed genes in the transcriptome; (C) radar plot of go enrichment of stress-related differentially expressed genes; (D) ROS levels in NTUH-K2044 and Φ-R NTUH-K2044; (E) expression levels of antioxidant-related genes in NTUH-K2044 and Φ-R NTUH-K2044; (F) oxidative stress resistance results in NTUH-K2044 and Φ-R NTUH-K2044.

Transcriptomic analyses of hvKp before and after phage treatment revealed extensive gene expression alterations, with 1341 genes significantly upregulated and 1088 downregulated, demonstrating (|log_2_FC| ≥ 1, FDR < 0.05) (Figure 4(A)). Gene Ontology (GO) enrichment of differentially expressed genes highlighted significant changes predominantly in regulatory processes, energy metabolism, and stress response, specifically oxidative stress (Figure 4(B,C)). ROS measurements revealed lower reactive oxygen species level in Φ-R NTUH-K2044, with expressions of ROS-detoxifying genes, *soxS* and *katE*, increasing by more than two-fold (Figure 4(D,E)). Meanwhile, expressions of *oxyR* and *katG* did not exhibit a two-fold change (Figure 4(E)). Oxidative stress resistance was further demonstrated in hydrogen peroxide survival assays; Quantitative survival data further supported enhanced oxidative stress tolerance: at 0.4 mM H_2_O_2_, survival of Φ-R NTUH-K2044 was 97.83 ± 21.43%, markedly higher than 0.283 ± 0.048% in NTUH-K2044; at 0.8 mM, the survival was 1.02 × 10^−4^ ±1.86 × 10^−5^ % versus 7.24 × 10^−6^ ±2.97 × 10^−6^ % (*n* = 6) (Figure 4(F)). Φ-R NTUH-K2044 showed higher survival rates than NTUH-K2044 at hydrogen peroxide concentrations of 0.4 mM and 0.8 mM, highlighting its enhanced oxidative stress resilience. To further explore regulatory dynamics, we examined upstream transcriptional regulators. Consistent with the two-step SoxRS system, *soxS* was upregulated while *soxR* was downregulated. Other stress- and capsule-associated factors showed varied responses: *rpoS* was decreased, hns remained stable, and *kvrA* expression was increased (S6 Fig). Given the K-locus deletions in Φ-R NTUH-K2044, this *kvrA* upregulation likely reflects compensatory or nonproductive regulation rather than a restoration of capsule synthesis.

### Compromised autophagy in host cells and enhanced intracellular survival of Phage-Resistant K. pneumoniae: cellular mechanisms unveiled

**Figure 5.**
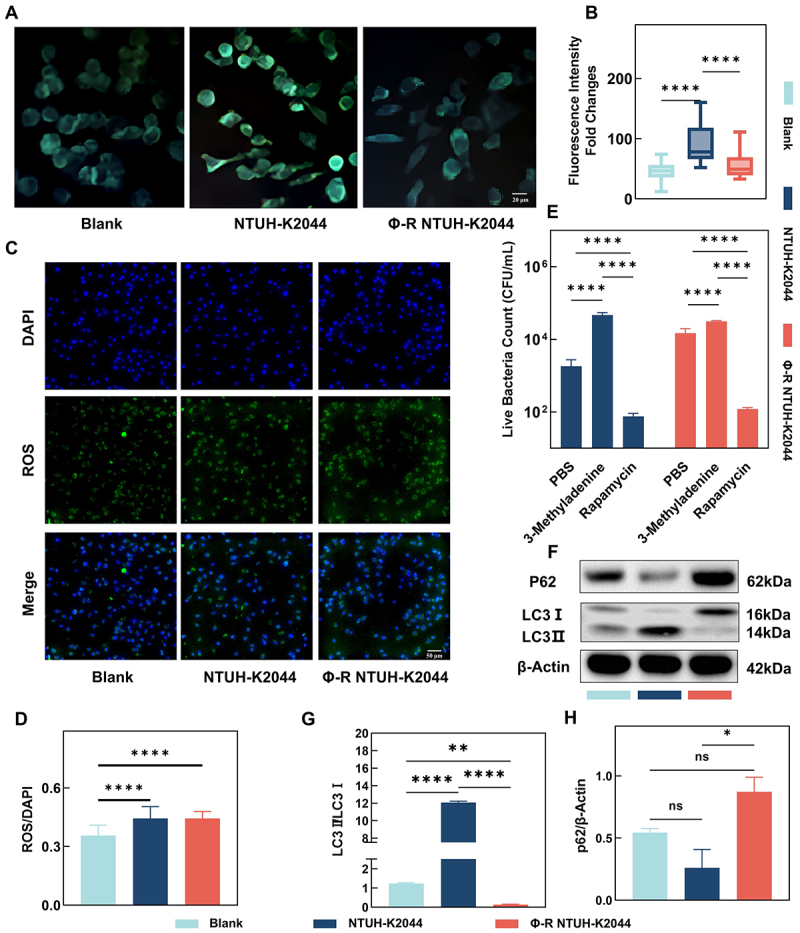
Macrophage intracellular clearance response to NTUH-K2044 and Φ-R NTUH-K2044. (A) autophagy visualization using MDC staining; (B) quantitative autophagy fluorescence intensities (ImageJ analysis); (C) ROS staining visualized under fluorescence microscopy; (D) quantitative analysis of ROS levels (ImageJ analysis); (E) impact of autophagy modulators on the survival of NTUH-K2044 and Φ-R NTUH-K2044 within macrophages; (F) Western blot analysis of autophagy-related proteins; (g) quantitative results for LC3 protein (ImageJ analysis); (H) quantitative results for P62 protein (ImageJ analysis).

**Figure 6.**
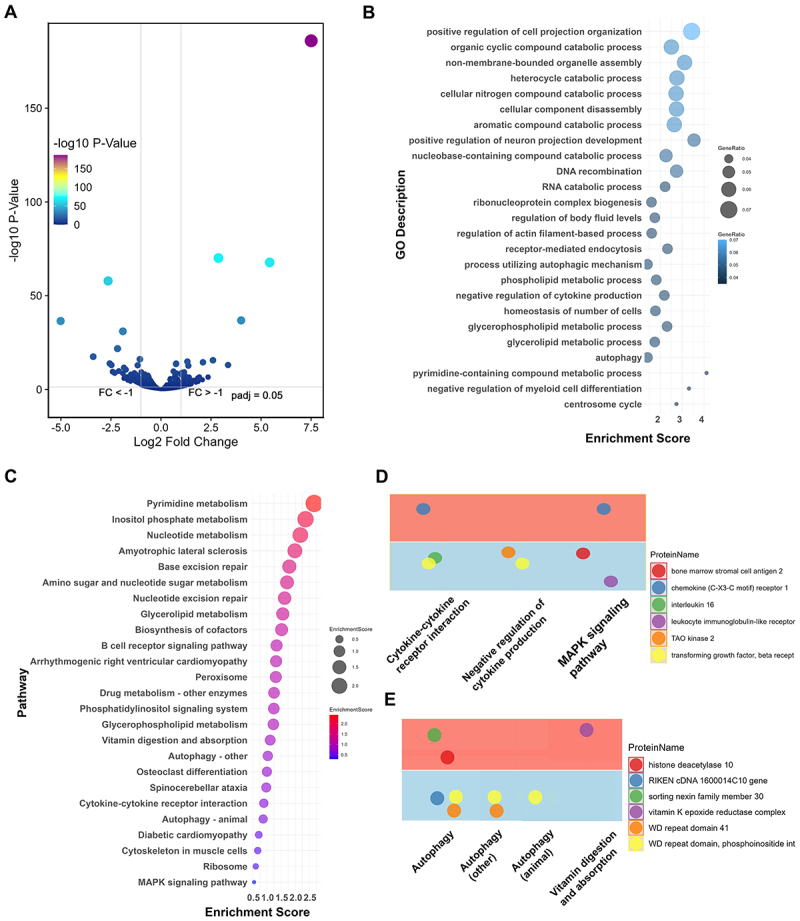
Protein expression changes in macrophages treated with Φ-R NTUH-K2044, compared to those treated with NTUH-K2044. (A) volcano plot illustrating the differential expression of proteins; (B) bubble charts of go enrichment analysis for differentially expressed proteins; (C) bubble charts showing KEGG pathway enrichment analysis for differentially expressed proteins; (D) expression levels within inflammation-related pathways; increases are shown in red areas, decreases in blue; (E) autophagy-related pathways expression levels; red indicates increased expression, blue indicates decreased.

Autophagic response, as revealed by MDC staining and quantified using ImageJ, showed that Φ-R NTUH-K2044 induced significantly lower fluorescence intensity compared to NTUH-K2044, with levels approaching those of uninfected controls (Figure 5(A,B)). Reactive oxygen species (ROS) assays indicated that although ROS levels increased post-infection compared to controls, there were no significant differences between cells infected with Φ-R NTUH-K2044 and those infected with NTUH-K2044 (Figure 5(C,D)). Additionally, confocal microscopy results using cholera toxin B subunit to label lipid rafts revealed no significant differences in lipid trafficking between cells treated with Φ-R NTUH-K2044 and those treated with NTUH-K2044 (S4A-S4B Fig). Investigations using autophagy modulators significantly altered the number of viable intracellular bacteria (Figure 5(E)), with no direct impact on bacterial proliferation (S5 Fig). Further analysis indicated that expression levels of autophagy-related genes in macrophages treated with Φ-R NTUH-K2044 were significantly lower than those treated with NTUH-K2044 (S4C Fig). Subsequently, Western blotting revealed a lower LC3-II/I ratio in Φ-R NTUH-K2044–infected macrophages compared to those infected with NTUH-K2044 (Figure 5(F,G)), indicating reduced levels of autophagy-associated markers. In contrast, p62 levels were decreased in NTUH-K2044–infected cells but remained relatively higher in Φ-R NTUH-K2044–infected macrophages (Figures. 5(F-H)). Taken together with the LC3-II/I ratio and MDC staining results, these findings are consistent with attenuated autophagy activity following Φ-R NTUH-K2044 infection. Proteomic analysis of RAW 264.7 cells treated with Φ-R NTUH-K2044, compared to those treated with NTUH-K2044, revealed significant alterations; 116 proteins showed significant changes in expression (|log_2_FC| ≥ 1, FDR < 0.05; Benjamini – Hochberg), with 68 upregulated and 48 downregulated (Figure 6(A)). Gene ontology enrichment analysis highlighted that these differentially expressed proteins were involved in diverse functions such as regulation of cellular components, metabolic processes, nucleic acid handling, autophagy, immune responses, and cell proliferation (Figure 6(B)). Concurrent KEGG pathway analysis indicated these differentially expressed proteins are implicated in metabolic processes, nutrient absorption, synthesis of cellular energy and building blocks, signal transduction, gene repair, DNA protection, cell differentiation, biosynthesis, and autophagy response to stress (Figure 6(C)). Further analysis focused on pathways related to immune responses and autophagy demonstrated distinct profiles; pathways such as cytokine-cytokine receptor interaction, negative regulation of cytokine production, and MAPK signaling pathway showed a notable decrease in inflammatory proteins like interleukin 16, bone marrow stromal cell antigen 2, leukocyte immunoglobulin-like receptor, and transforming growth factor-beta receptor, except for an increase in chemokine (C-X3-C motif) receptor 1 (Figure 6(D)). Notably, autophagy-related pathways, including general autophagy, other autophagy, and autophagy in animals, along with vitamin digestion and absorption, showed an upregulation of proteins like sorting nexin family member 30, histone deacetylase 10, and vitamin K epoxide reductase complex, while proteins such as the RIKEN cDNA 1600014C10 gene, WD repeat domain 41, and WD repeat domain, phosphoinositide int showed a decrease (Figure 6(E)).

### Discussion

Intracellular hypervirulent *Klebsiella pneumoniae* (hvKp) pose a significant challenge due to their ability to disseminate rapidly through macrophages across tissues, leading to severe bloodstream infections [3,6]. Moreover, their intracellular lifestyle enhances antibiotic resistance, complicating treatment as drugs struggle to penetrate and clear the bacteria effectively [12,13,22,23]. In this study, intracellular NTUH-K2044 exhibited increased antibiotic tolerance, surviving kanamycin treatment at concentrations as high as 50 µg/mL due to limited intracellular penetration of the antibiotic (Figure 1(B), 2(D)). The unique ability of phages to penetrate cellular barriers and exert antimicrobial effects makes them a promising approach for combating intracellular hvKp [18,24]. However, our findings indicate that phage-resistant hvKp can also emerge within host cells, similar to extracellular counterparts [25,26]. Notably, although this capsule-deficient resistant strain is more readily phagocytized by macrophages similar to typical *K. pneumoniae*, unlike typical *K. pneumoniae*, it exhibits unexpectedly enhanced intracellular survival. While not immediately lethal, this strain still inflicts significant damage on host tissues and organs. This reveals potential deficiencies in the current phage therapy strategy to effectively address intracellular hvKp infections.

Phage therapy significantly reduced the mortality of our intracellular hvKp infected mouse model (Figure 1(C)), suggesting its potential as an anti-virulent agent. However, using mortality alone to assess phage therapy might be insufficient, as it does not fully capture the complexity of infection dynamics and organ damage. As observed in this study, the phage therapy did not significantly improve the structural integrity of organs, which might be correlated with bacterial loads (Figure 1(D)). In support of this, peritoneal immune cells from mice treated with phages were found to contain greater quantities of intracellular bacteria (Figure 1(B)), and subsequent bacterial counts confirmed increased bacterial loads in organs of mice infected with phage-treated intracellular bacteria (Figure 1(E)). Our data suggest that the observed survival benefit stems primarily from reduced immunopathology rather than improved bacterial clearance. The phage-selected, capsule-deficient variant elicited markedly lower pro-inflammatory cytokine production (IL-1β, TNF-α, IL-6; Figure 3(D-F)) and attenuated release of other inflammatory mediators (Figure 6(D)), despite higher tissue bacterial burdens (Figure 1(B–E)). Concurrently, Φ-R NTUH-K2044 exhibited enhanced oxidative-stress resilience (elevated *soxS*/*katE* expression and improved H_2_O_2_ survival; Figure 4(D-F)) and reduced autophagy-associated responses (as indicated by MDC staining, LC3-II/I ratio, and p62 levels; Figure 5(A,B,F-H)), without an increase in host-derived ROS (Figure 5(C,D)). Collectively, these findings suggest that diminished host tissue damage and specific bacterial adaptations may uncouple mortality from pathogen burden in this infection model. The higher bacterial burden observed in our study, it did not necessarily result in increased mortality, echoing the notion that inflammatory cytokine production and inflammation, rather than the bacterial load per se, are the pivotal lethality factors in infectious diseases [27,28]. Consistently, our findings reveal reduced levels of cytokines such as IL-1β, TNF-α, and IL-6 in mice infected with Φ-R NTUH-K2044 (Figure 3(D–F)), which corresponded to lower mortality rates compared to mice infected with NTUH-K2044. The strong inverse correlation between early inflammatory cytokine levels and day7 survival supports the interpretation that phage therapy may improve prognosis at least in part by attenuating excessive inflammation. Proteomic analysis supports this, showing decreased levels of inflammatory mediators like interleukin 16 and transforming growth factor-beta receptor (Figure 6(D)), elucidating the mechanisms of cytokine modulation. Φ-R NTUH-K2044 exhibited a stable phenotype of capsule loss (Figure 3(A)), due to deletions in the capsule coding regions (Figure 3(C)). Both the phenotype and its genetic basis mirror the extracellular resistance documented in our team’s previous publication [29]. This capsule loss phenotype contributes to reduced viscosity and virulence, aligning with the observed reductions in mortality (Figure 3(D)) [30,31]. Although the capsule is known to aid *K. pneumoniae*‘s resistance to macrophage phagocytosis and intracellular survival, our findings are consistent with this view, as intracellular hvKp obtained from phage-treated mice, characterized by capsule loss (Φ-R NTUH-K2044), exhibited increased phagocytosis by macrophages compared to intracellular hvKp from untreated mice (NTUH-K2044) [32,33]. However, despite this increased phagocytosis, intracellular hvKp from phage-treated mice demonstrated unexpectedly enhanced survival and proliferation within macrophages, indicating the possibility of compensatory mechanisms need to be explored. Subsequent transcriptomic analyses to explore potential factors supporting the enhanced survival of Φ-R NTUH-K2044 revealed shifts in metabolic and stress response pathways (Figure 4(B)), which may play a role in enhancing intracellular survival of Φ-R NTUH-K2044. However, growth curves indicated that, despite these metabolic changes, Φ-R NTUH-K2044 did not exhibit enhanced growth characteristics compared to NTUH-K2044. (S5 Fig). Given that oxidative stress is a crucial bactericidal mechanism of macrophages, the significant upregulation of *soxS* and *katE* genes in Φ-R NTUH-K2044, which enhances its resistance to oxidative stress, was pivotal (Figure 4(D–E)) [3]. Φ-R NTUH-K2044 showed increased resistance to oxidative stress (Figure 4(F)), without a corresponding increase in oxidative stress level from macrophages (Figure 5(C,D)), suggesting that the enhanced survival of Φ-R NTUH-K2044 within macrophages may be attributed to intrinsic bacterial adaptations.

Analysis from the perspective of intracellular hvKp is crucial, and similarly, understanding its adversaries in infection scenarios, such as immune cells and phages, is equally important. Both intracellular NTUH-K2044 and intracellular Φ-R NTUH-K2044 led to a significant reduction in white blood cell counts of infected mice (Figure 2(A)), indicating severe systemic infections. However, infection from intracellular Φ-R NTUH-K2044 showed significantly lower macrophage depletion compared to that from NTUH-K2044 (Figure 2(A)). Further studies revealed that Φ-R NTUH-K2044 proliferates at a higher rate within macrophages (Figure 2(E)), suggesting an adaptation that enhances its survival capability. Regarding the role of phages, they are generally known to be ineffective against phage-resistant bacteria. However, while some studies suggest that phages might promote bacterial clearance by immune cells, others report no interaction or even an inhibitory effect on immune cell activity [34,35]. These observations indicate that the effects of a phage can vary significantly under different experimental conditions and environments, underscoring the need for a more systematic investigation to fully understand its specific impacts across various conditions. Consequently, we investigated the role of the phage used in our study, and our findings suggest that it did not significantly affect the bactericidal activity of macrophages. (Figure 2(A), 3(E,F), S2 and S3). Moving forward, our research will focus on exploring the response of immune cells, aiming to elucidate how they interact with and respond to intracellular hvKp. Despite macrophages phagocytosing more Φ-R NTUH-K2044 (Figure 2(C)), a higher number of it ultimately survived after 24 hours (Figure 2(D)), contrary to the intent of antimicrobial treatment. ROS and autophagy are crucial defense mechanisms employed by macrophages to combat intracellular bacteria, playing key roles in controlling bacterial infections [36,37]. Although ROS levels in macrophages treated with both NTUH-K2044 and Φ-R NTUH-K2044 were elevated compared to controls, no significant difference was observed between them (Figure 5(C,D)). However, compared with NTUH-K2044, autophagy-associated markers – including MDC fluorescence intensity, LC3-II/I ratio, and p62 levels – were consistently lower or shifted in a pattern suggestive of reduced autophagy activity during Φ-R NTUH-K2044 infection (Figure 5(A,B, F–H)). Several studies have highlighted the role of autophagy in modulating intracellular bacterial populations [38,39]. In this study, the reduction in autophagic activity is directly implicated in the enhanced survival of *K. pneumoniae* within macrophages. We specifically demonstrated this relationship through our experiments with autophagy modulators, which significantly altered the intracellular persistence of *K. pneumoniae*, as evidenced by our results (Figure 5(E)). This direct modulation of autophagy underscores its critical role in facilitating hvKp survival inside host cells. To investigate the mechanisms of autophagy alterations directly associated with the intracellular survival of hvKp, we compared the proteomic profiles of RAW264.7 cells treated with Φ-R NTUH-K2044 and NTUH-K2044. The analysis revealed significant changes in proteins involved in inflammation and autophagy pathways in macrophages treated with Φ-R NTUH-K2044 compared to those treated with NTUH-K2044 (Figure 6(A–C)). Notably, autophagy-associated protein TAO kinase 2 showed decreased expression levels, which may be associated with reduced autophagic activity [40,41]. Similarly, the downregulation of WD repeat domain 41 proteins and phosphoinositide interacting proteins, crucial for autophagy initiation and autophagosome formation, may also contribute to diminished autophagic activity [42–45]. Additionally, the upregulation of vitamin K epoxide reductase complex protein, involved in the vitamin digestion and absorption pathway, might accelerate the degradation of vitamin K produced by *K. pneumoniae* [46]. This decrease in vitamin K levels could reduce vitamin-induced autophagy in macrophages, thereby impacting their ability to clear intracellular bacteria [47,48]. In summary, compared to macrophages infected with NTUH-K2044, macrophages infected with Φ-R NTUH-K2044 exhibited weaker autophagic effects. Based on our measurements, these differences could not be attributed to changes in GM1-enriched membrane structures (CTB staining; Fig. S4A – B) or ROS levels (Figure 5(C,D)) in macrophages [49–51]. Interestingly, we observed changes in autophagy-associated proteins such as TAO kinase 2, WD repeat domain proteins, and the vitamin K epoxide reductase complex, indicating alterations in autophagy regulation [52,53]. These findings shed light on autophagy regulation within macrophages, revealing notable differences in response to Φ-R NTUH-K2044 compared to NTUH-K2044. However, the underlying molecular mechanisms are still not fully understood due to the limitations of our current experimental design and resources. Future studies are necessary to precisely define these roles and assess their clinical potential.

This study confirms that phage therapy can significantly reduce mortality from intracellular infections by hypervirulent Klebsiella pneumoniae, underscoring its potential as a targeted anti-virulence strategy. Crucially, we observed that hvKp strains develop resistance to phages, enhancing their survival by attenuating key macrophage antimicrobial pathways, including inflammation and autophagy. Such adaptations enable these resistant strains to undermine therapeutic efficacy and facilitate systemic infection, acting akin to a “Trojan horse”. Notwithstanding these adaptive risks, phages remain an important option for acute infections, especially when used in combination regimens – such as phage – antibiotic therapy, multi-receptor cocktails, or host-directed addons – that can help constrain resistance and reinforce intracellular killing. These findings highlight the critical need for ongoing research to optimize phage therapy, particularly through understanding and manipulating the complex host-pathogen interactions within intracellular environments.

## Materials and methods

### Bacterial strains, phage, Eukaryotic cells, and culture conditions

The hvKp strain NTUH-K2044 purchased from ATCC was cultured overnight in Luria-Bertani (LB) medium from −80°C cryopreserved stocks. Phage ΦK2044, belonging to the *Autographiviridae* family, was isolated from wastewater and maintained in our laboratory [29]. RAW264.7 murine macrophage cells were revived from cryopreserved stocks in DMEM. Cells were used at passage 12. ΦK2044 was resuscitated using NTUH-K2044 suspensions. For *in vitro* co-culture, RPMI 1640 medium with 10% fetal bovine serum was used to assess bacteria – macrophage – phage interactions [54].

### Growth curve analysis

Overnight *K. pneumoniae* cultures were diluted to an initial OD_600_ of 0.01. Cultures were treated with 5 nM 3-Methyladenine or 12.5 nM Rapamycin, then incubated at 37°C with 180 rpm shaking for 24 hours. OD_600_ was measured hourly to monitor growth.

### MOI-Dependent phage resistance assay [55]

To assess phage-resistant hvKp formation at varying MOIs, 180 μL of NTUH-K2044 suspension (10^7^ CFU/mL) was added to a 96-well plate, followed by 20 μL of phage to achieve MOIs of 0, 0.001, 0.01, 0.1, and 1. Plates were incubated with shaking at 180 rpm, and OD_600_ was measured at 0, 1, 2, 3, 4, 5, 6, 7, 8, 9, 10, 11, 12, and 24 hours using a spectrophotometer.

### Micro-drop Colony Counting

Sterile eight-well strips were placed on a PCR plate, and 180 µL of sterile PBS was added to each well. A 20 µL bacterial suspension was serially diluted by transferring 20 µL between wells, achieving dilutions from 10^0^ to 10^−8^. From each dilution, 10 µL was spotted in triplicate onto agar plates. Plates were incubated at 35°C, and colonies were counted the next day from spots containing 3–30 colonies.

### Two-stage intracellular hvKp infection murine model

Animal experiments were approved by the Ethics Committee of The First Hospital of Wenzhou Medical University (Approval No.: WYYY-AEC-2022–047), in accordance with the ‘Wenzhou City Experimental Animal Welfare and Ethical Standards.” Experiments involved female C57BL/6C mice (Charles River Laboratories, Massachusetts, USA), aged 6 to 8 weeks, following slightly modified standard procedures [9]. In stage one, mice received a peritoneal injection of 2 × 10^4^ CFU hvKp, immediately followed (within 5 min) by a single intraperitoneal injection of 10 µL PBS (control) or 10 µL phage suspension (2 × 10^3^ phages, MOI 0.1); no additional phage administrations were performed. The Stage one intraperitoneal inoculum (2 × 10^4^ CFU) was chosen based on pilot titrations to reliably establish intracellular hvKp while minimizing early lethality and excessive peritoneal inflammation. At 24 h post-infection, mice were euthanized by CO_2_ asphyxiation with cervical dislocation, and peritoneal cells were collected via cold PBS rinses, centrifuged at 200 g for 5 minutes, and treated with 50 µg/mL kanamycin for 30 minutes to remove extracellular bacteria. Cells were lysed with 0.2% Triton X-100, and intracellular bacteria were quantified using the Micro-drop Colony Counting method. In stage two, pooled peritoneal cells containing intact intracellular bacteria were prepared, and the number of intracellular bacteria was adjusted to approximately 8 × 10^3^ CFU per mouse prior to intravenous injection via the tail vein, without lysing the cells. Before Stage two transfer, peritoneal cell suspensions were washed three times with cold PBS and resuspended in fresh PBS to minimize freephage carryover. The Stage two intravenous dose (8 × 10^3^ CFU per mouse) was then normalized to the number of viable intracellular bacteria recovered at 24 h, ensuring a comparable bloodstream challenge across groups while preserving cellassociated delivery. Mice were then monitored daily for survival over seven days, with final euthanasia by CO_2_ asphyxiation and cervical dislocation on day 7.

### Hematological analysis and tissue processing

Orbital blood samples were collected from anesthetized mice 48 hours post-intracellular bacterial infection into heparinized tubes. Blood cell counts and hematology profiles were analyzed using a HemaVet 950 FS, with reference values from Charles River Laboratories. Vital organs were harvested for histological and bacteriological analysis. One portion of each organ was fixed, embedded, and stained with hematoxylin and eosin for histological examination, while the other was processed for bacterial load determination via Micro-drop Colony Counting.

Pathology scoring, tailored to each organ type, was conducted by two independent pathologists to ensure reliability. Scoring criteria evaluated infection-related changes, including myocardial inflammation, hepatocellular damage, renal tubular damage, and alveolar congestion [56–59]. Detailed scoring systems are available in S1-S4 Table.

### Genomic characterization and analysis

Whole-genome sequencing of *K. pneumoniae* was performed using the Illumina HiSeq platform. Genomes were assembled with Unicycler (v0.5.0) and genetic variations were aligned to the NTUH-K2044 reference genome (AP006725.1) using Breseq (v0.38.1). Results were visualized with Easyfig (v2.2.5).

### Transcriptomic profiling

NTUH-K2044 cultures at 8 hours post-infection served as the control for comparison with Φ-R NTUH-K2044 at the same time point. Total RNA was extracted with the Qiagen RNeasy Mini Kit and verified (RIN > 7.0) using an Agilent Bioanalyzer. RNA-seq libraries were prepared with the NEBNext Ultra II Directional RNA Library Prep Kit for Illumina and sequenced on the Illumina HiSeq 2500 platform. Bioinformatics analysis included read mapping (Hisat2), gene expression quantification (FeatureCounts), and differential expression analysis (DESeq2). Further analyses included GO enrichment (gProfiler), network visualization (Cytoscape), and custom R scripts for pathway analysis.

### Quantitative real-time PCR

RNA was isolated, followed by cDNA synthesis and real-time PCR using SYBR Green chemistry. Relative gene expression was quantified using the 2^−ΔΔct^ method. Primer sequences are detailed in S5 Table.

### Transmission electron microscopy (TEM)

TEM procedures were conducted with slight modifications from previously described methods [60]. RAW264.7 cells co-cultured with bacteria for 24 hours were fixed with 2% glutaraldehyde and paraformaldehyde in sodium cacodylate buffer (pH 7.2) for 3 hours at room temperature, then overnight at 4°C. Postfixation was performed with 1% osmium tetroxide for 1 hour, followed by dehydration through graded ethanol and embedding in epoxy resin for 16 hours. Ultrathin sections were stained with 2% uranyl acetate and lead citrate, and images were captured using a Hitachi H-7650 transmission electron microscope.

### Western blot

Experimental methods were adapted from previously published studies with slight modifications [61]. Briefly, RAW 264.7 cells were co-cultured with hvKp at a ratio of 1:100 for 6 hours, followed by 50 µM chloroquine treatment for 2 hours to monitor autophagy markers. Proteins were extracted using the Soluble & Insoluble Protein Extraction Kit (Biorab, GS1461), separated via SDS-PAGE, and transferred to PVDF membranes (Millipore, USA). Membranes were probed with primary antibodies for LC3B (Cell Signaling, #2775), SQSTM1/p62 (Abcam, ab109012), and β-Actin (Abcam, ab8226), and visualized using the QuickChemi 5100 Chemiluminescence Imaging System.

### Reactive oxygen species detection

Overnight LB cultures of *K. pneumoniae* were washed three times with PBS, diluted to OD_600_ 0.3 – 0.4, and stained with 10 μM DCFH-DA (Beyotime Biotechnology, Shanghai, China) at 37°C for 1 hour in the dark. After three washes with PBS, fluorescence was measured using a microplate reader (excitation at 488 nm, emission at 520 nm).

### Enzyme-linked immunosorbent assay for cytokine measurement

IL-6 (BP-E20012), IL-10 (BP-E20005), IL-1β (BP-E20533), IL-18 (BP-E200001), and TNF-α (BP-E20220) were quantified using commercial ELISA kits following the manufacturer’s instructions. RAW264.7 cells were incubated with PBS or *K. pneumoniae* isolates for 6 hours. Supernatants were collected post-centrifugation (12,000 rpm, 5 min) and analyzed per kit guidelines. We derived an Inflammation-Score by calculating the arithmetic mean of the z-scores of log_1__0_-transformed concentrations of IL-1β, TNF-α, IL-6, and IL-18. IL-10 was excluded a priori due to its anti-inflammatory nature and inverse regulation, in order to retain a unidirectional inflammatory construct. These replicate-level scores were then correlated with day-7 survival proportions at the group level using a two-sided Spearman’s rank correlation.

### Confocal microscopy

RAW264.7 cells were plated in 24-well plates and incubated overnight at 37°C in 5% CO_2_. Cells were treated with PBS or *K. pneumoniae* isolates. For autophagy, cells were stained with Monodansylcadaverine (MDC) for 30 min at 37°C. ROS detection involved staining with 10 μM DCFH-DA for 1 hour at 37°C. Lipid trafficking was assessed by fixing cells in 4% paraformaldehyde and staining with Alexa594-conjugated cholera toxin B subunit (Alexa594-CTB) for 30 min at 4°C. Following treatments, cells were counterstained with DAPI, visualized using a Nikon ECLIPSE Ti2 confocal microscope at 400× magnification, and analyzed with ImageJ.

### Phagocytosis and intracellular survival assay

The intracellular survival assay was conducted according to previously established methods with slight modifications [8]. Briefly, were plated in 24-well plates and incubated overnight at 37°C in 5% CO_2_. Bacteria were added at a 100:1 ratio in 500 μl antibiotic-free medium. Plates were centrifuged at 200 g for 5 min to synchronize infections, followed by a 2-hour incubation. Cells were then washed and treated with 50 μg/ml kanamycin for 30 min to eliminate extracellular bacteria. For the phagocytosis assay, cells were lysed with 0.2% Triton X-100 at 37°C for 20 min. For intracellular survival, cells were incubated in fresh medium for 22 hours post-kanamycin treatment before lysis. Intracellular bacterial loads at 2 and 24 hours were determined using the Micro-drop Colony Counting method. Experiments were conducted in duplicate across three independent runs.

### Mucoviscosity assessment

Mucoviscosity was assessed using the string test on Columbia agar plates, with colonies stretchable to at least 5 mm considered positive. For the sedimentation assay, cultures normalized to an OD_600_ of 1 were centrifuged at 1000 × g for 5 min, and supernatant clarity was observed.

### Acute peritoneal infection mice model

Specific Pathogen-Free (SPF) male ICR mice (aged 4–5 weeks, Charles River Laboratories) were intraperitoneally injected with 200 µL of bacterial suspension (2 × 10^5^ CFU). Survival was monitored daily for seven days to evaluate lethality.

### H_2_O_2_ survival assessment

The H_2_O_2_ survival test was modified from previous methods [3]. Bacterial aliquots were exposed to 0, 0.2, 0.4, and 0.8 mM H_2_O_2_ at 37°C for 4 hours. Viable bacteria were quantified using the Micro-drop Colony Counting method.

### Proteomics sequencing and analysis

RAW 264.7 cells co-cultured with hvKp (1:100 ratio, 6 hours) were lysed using SDT buffer (4% SDS, 100 mM Tris-HCl, pH 7.6). Protein concentration was determined via the BCA Protein Assay Kit. Proteins were separated by SDS-PAGE and digested using the Filter-Aided Sample Preparation (FASP) technique. Peptides were cleaned on C18 cartridges, concentrated, and resuspended in 0.1% formic acid for LC-MS/MS analysis on a Bruker timsTOF Pro. T Data acquisition utilized a C18 column and acetonitrile gradient in 0.1% formic acid, operated in PASEF mode. Proteins were identified and quantified with MaxQuant software. Bioinformatics analysis included GO annotation (NCBI BLAST+, InterProScan), KEGG pathway mapping, and enrichment analysis with Fisher’s exact test and Benjamini-Hochberg correction. Visualizations were generated in R.

### Statistical analysis

Data are presented as mean ± standard deviation from at least three independent experiments. Statistical analyses were performed using Prism software with ANOVA (Bonferroni correction) or unpaired t-test. Statistical significance in figures is denoted as ns (*p* > 0.05), * (*p* < 0.05), ** (*p* < 0.01), *** (*p* < 0.001), **** (*p* < 0.0001). The study adhered to the ARRIVE guidelines.

## Supplementary Material

Clean Copy of Supplementary Legends- QVIR-2025-0342.R1.docx

S4 Table.docx

S5 Fig.jpg

S3 Table_revised.docx

S1 Fig.jpg

S5 Table.docx

S6 Fig_revised.jpg

S1 Table.docx

S2 Fig.jpg

S3 Fig.jpg

S4 Fig.jpg

S2 Table.docx

## Data Availability

The authors declare that all data supporting the findings of this study are available in the paper. The data set linked with this submission can be found at https://doi.org/10.57760/sciencedb.24611 [62]. Whole-genome sequencing (WGS) data for Φ-R NTUH-K2044 and transcriptomic data of NTUH-K2044 before and after phage treatment are available in the NCBI SRA under BioProject PRJNA1160486 (https://www.ncbi.nlm.nih.gov/bioproject/PRJNA1160486/) [63]. Proteomic raw data are available in iProX under accession IPX0009720000 (https://www.iprox.cn/page/project.html?id=IPX0009720000) [64].
