# Supplementary material for: Evolving strategies of intracellular Hypervirulent Klebsiella pneumoniae during phage therapy: Reducing host autophagy and inflammation
Source: Virulence. 2025 Dec 4;16(1):2600148. doi: 10.1080/21505594.2025.2600148 (PMC12688233; doi:10.1080/21505594.2025.2600148)
Supplement: Clean Copy of Supplementary Legends- QVIR-2025-0342.R1.docx [file KVIR_A_2600148_SM0650.docx]

**Supplemental information titles and legends**

S1 Fig. Impact of phage multiplicity of infection (MOI) on the development of phage resistance in hypervirulent *Klebsiella pneumoniae* (hvKp) NTUH-K2044.

S2 Fig. Evaluating phage impact on inflammatory cytokine production in macrophages. A: Gene expression levels of inflammatory factors (qRT-PCR); B: Expression levels of inflammatory factors (ELISA). Note on dosages: In this experiment, the phage dosage was 10 times the number of macrophages, with a phage-to-bacteria ratio (MOI) of 0.1 and a bacteria-to-macrophage ratio of 100.

S3 Fig. Role of phage exposure in modulating macrophage activity and survival of intracellular phage resistant *K. pneumoniae*. A: Bacterial phagocytose by mouse macrophages RAW264.7, 2 hours post-infection; B: Count of bacteria surviving within mouse macrophages RAW264.7, 24 hours post-infection; C: Growth ratio of intracellular bacteria in mouse macrophages RAW264.7 calculated as 24-hour survival relative to initial phagocytosis at 2 hours post-infection.

S4 Fig. Autophagy-associated membrane domains and gene expression in RAW264.7 cells. A: Confocal microscopy images of GM1-enriched lipid rafts visualized by cholera toxin B (CTB) staining. B: Quantitative analysis of lipid raft fluorescence intensity using ImageJ. C: Expression levels of autophagy-related genes (normalized to control).

S5 Fig. Growth curves of hvKp and phage-resistant hvKp following treatment with various autophagy modulators.

S6 Fig. Candidate upstream regulators in phage-driven hvKp adaptation (transcriptomics)

S1 Table. Scoring Criteria for Myocardial Injury Based on Histopathological Assessment.

S2 Table. Modified Ishak Score for Liver Histology Activity Index.

S3 Table. Lung Tissue Injury Scoring System.

S4 Table. Kidney Tissue Damage Scoring System.

S5 Table. qPCR primers for autophagy and inflammatory factor encoding genes.
