# Supplementary material for: Evolving strategies of intracellular Hypervirulent Klebsiella pneumoniae during phage therapy: Reducing host autophagy and inflammation
Source: Virulence. 2025 Dec 4;16(1):2600148. doi: 10.1080/21505594.2025.2600148 (PMC12688233; doi:10.1080/21505594.2025.2600148)
Supplement: S4 Table.docx [file KVIR_A_2600148_SM0649.docx]

S4 Table. Kidney Tissue Damage Scoring System

|  | Score | | | |
| --- | --- | --- | --- | --- |
|  | 0 | 1 | 2 | 3 |
| Tubular Atrophy | Normal | Mild damage | Moderate damage | Severe damage |
| Epithelial Cell Degeneration |  |  |  |  |
| Epithelial Cell Necrosis |  |  |  |  |
| Interstitial Edema |  |  |  |  |
| Interstitial Fibrosis |  |  |  |  |
| Inflammatory Cell Infiltration |  |  |  |  |

Cumulative scores range from 0 to 18, with total scores ≤ 4 indicating minor tubulointerstitial damage,

5-8 indicating moderate damage, and ≥ 9 indicating severe damage.
