# Supplementary figures and images for: Evolving strategies of intracellular Hypervirulent Klebsiella pneumoniae during phage therapy: Reducing host autophagy and inflammation
Source: Virulence. 2025 Dec 4;16(1):2600148. doi: 10.1080/21505594.2025.2600148 (PMC12688233; doi:10.1080/21505594.2025.2600148)

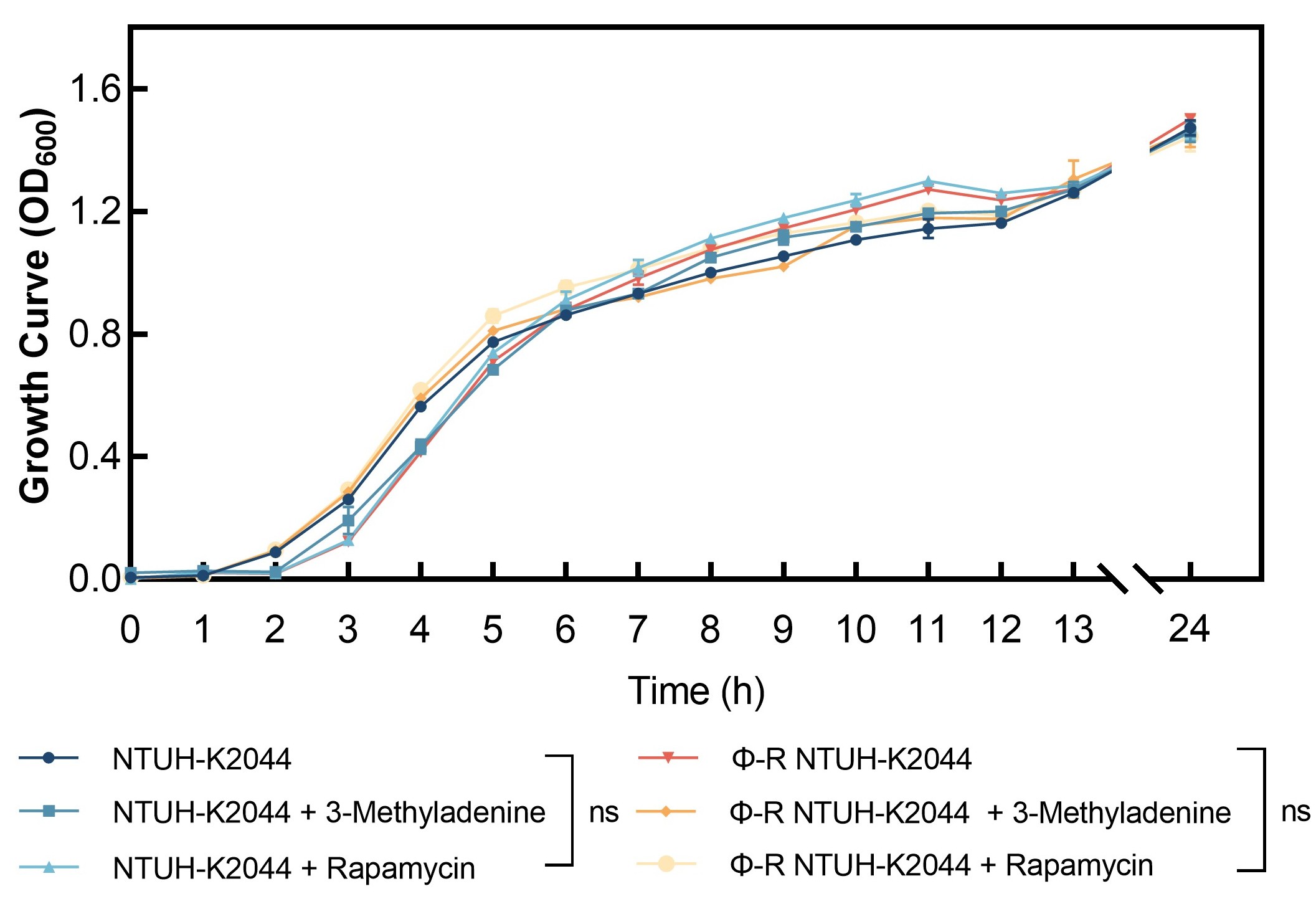

Supplement: S5 Fig.jpg [file KVIR_A_2600148_SM0648.jpg]

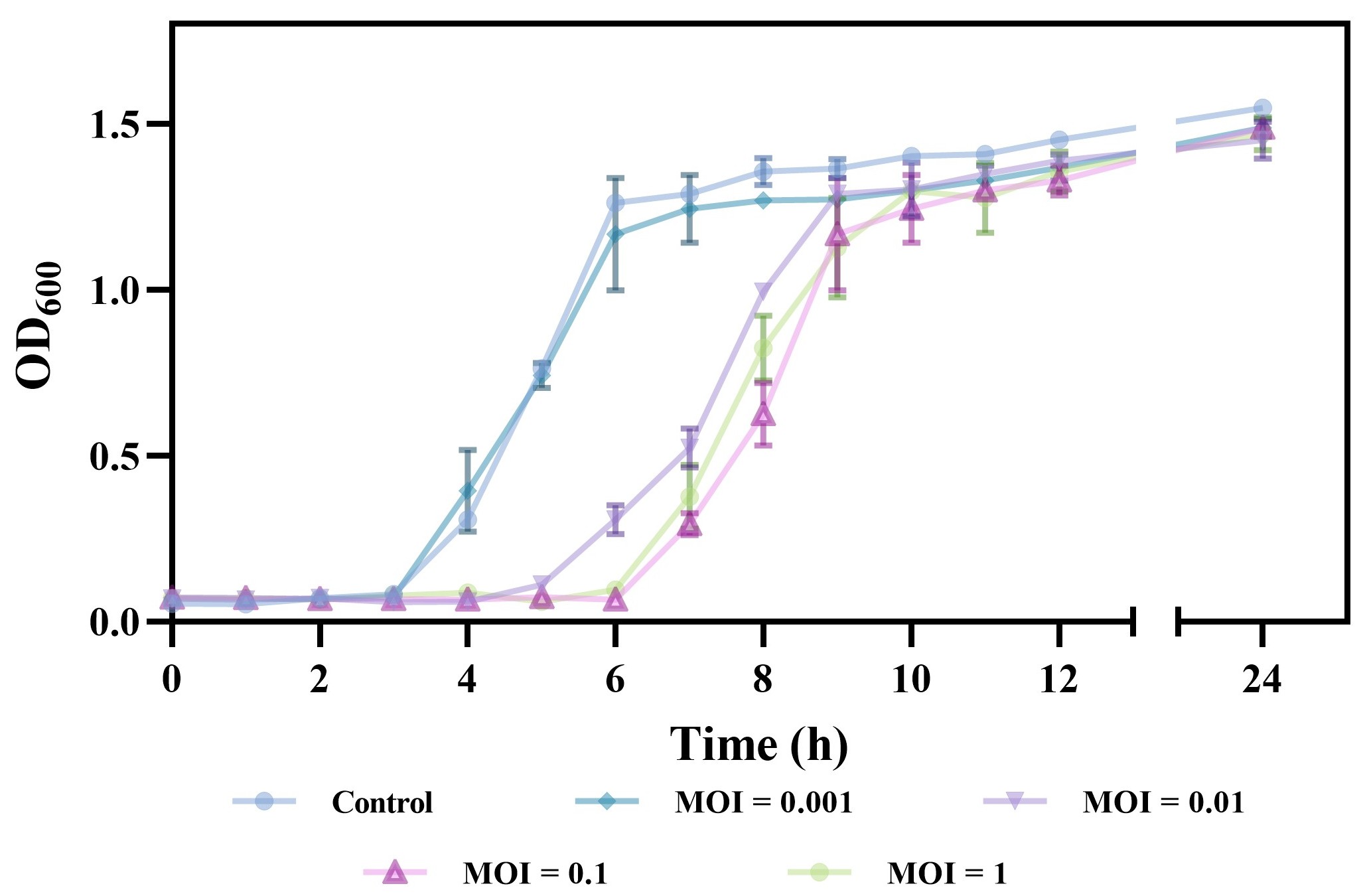

Supplement: S1 Fig.jpg [file KVIR_A_2600148_SM0646.jpg]

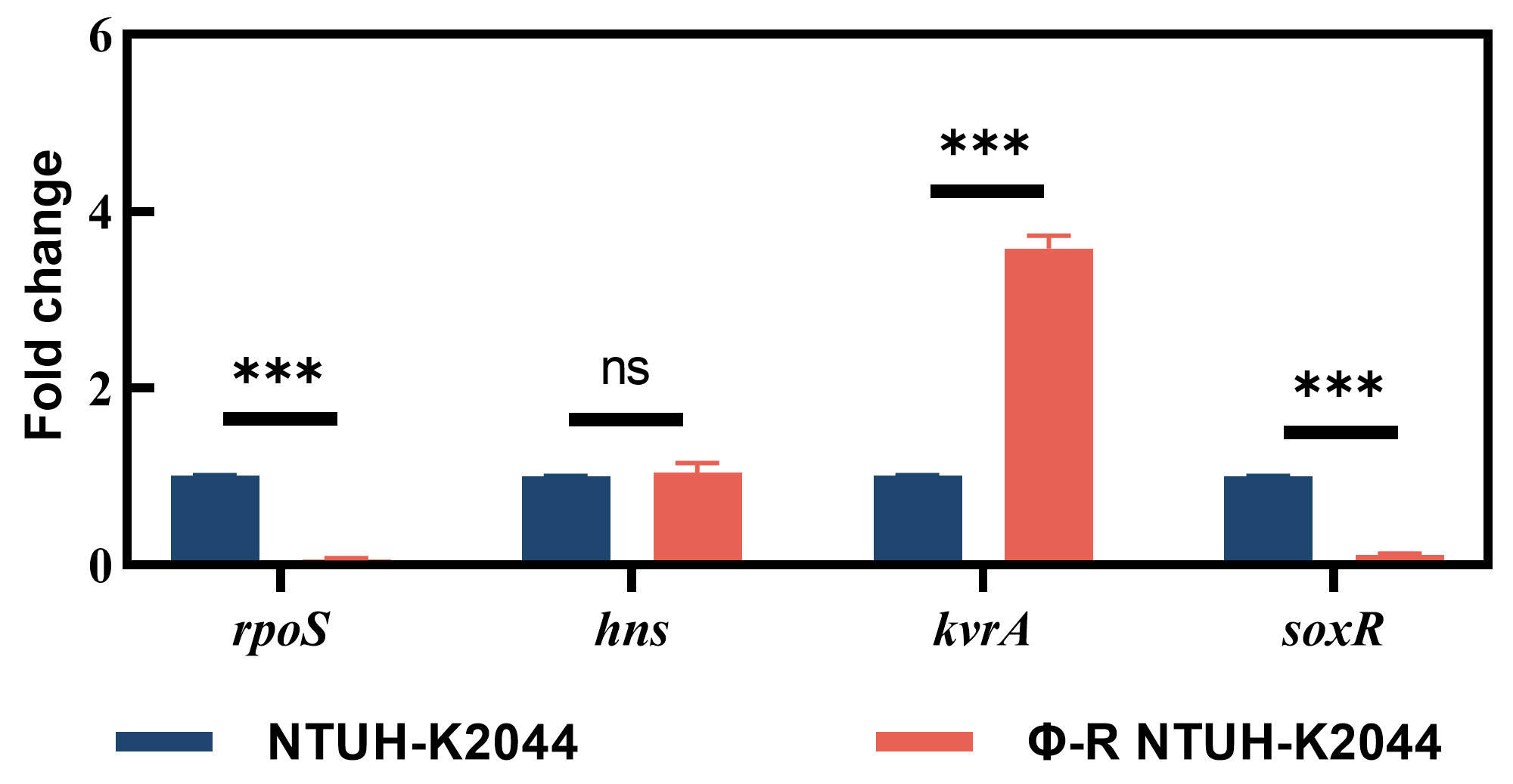

Supplement: S6 Fig_revised.jpg [file KVIR_A_2600148_SM0644.jpg]

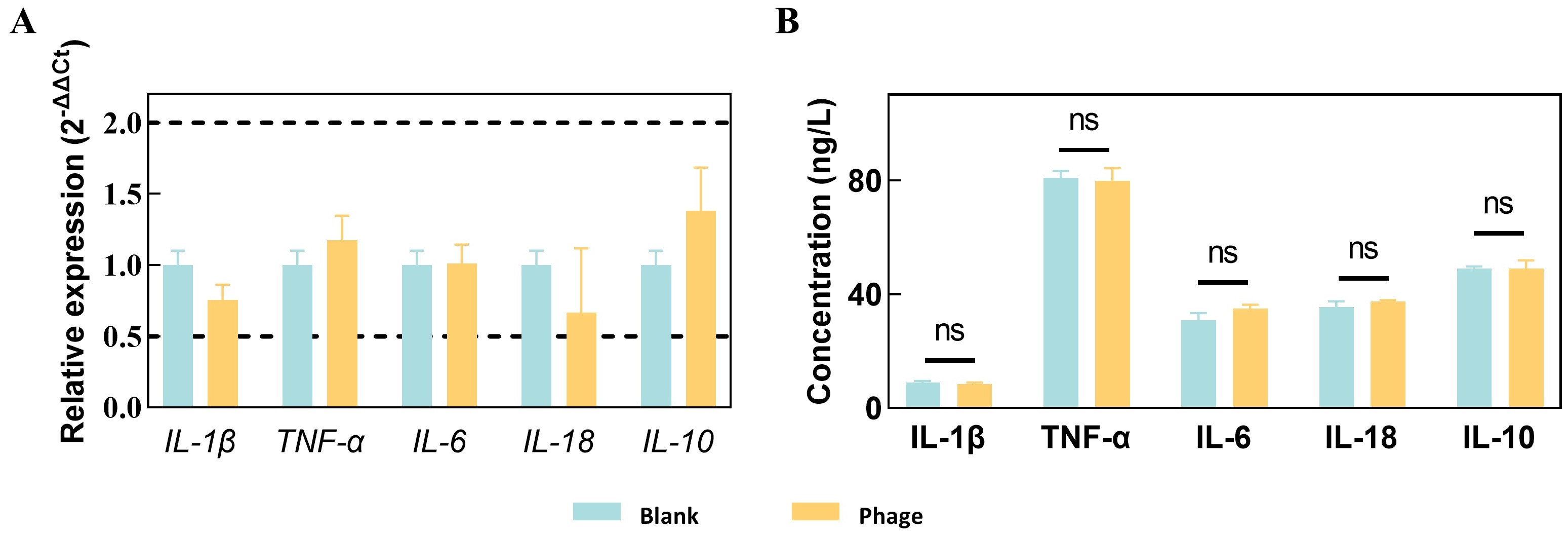

Supplement: S2 Fig.jpg [file KVIR_A_2600148_SM0642.jpg]

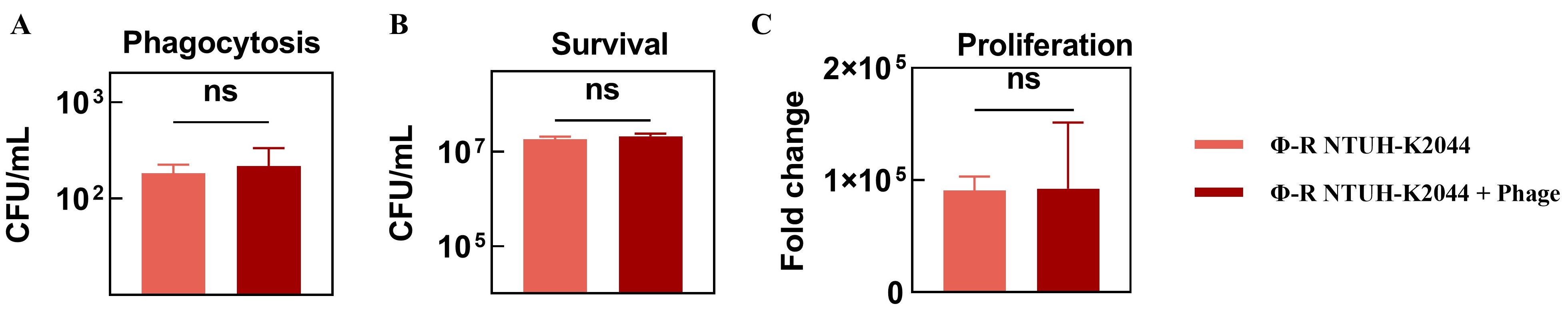

Supplement: S3 Fig.jpg [file KVIR_A_2600148_SM0641.jpg]

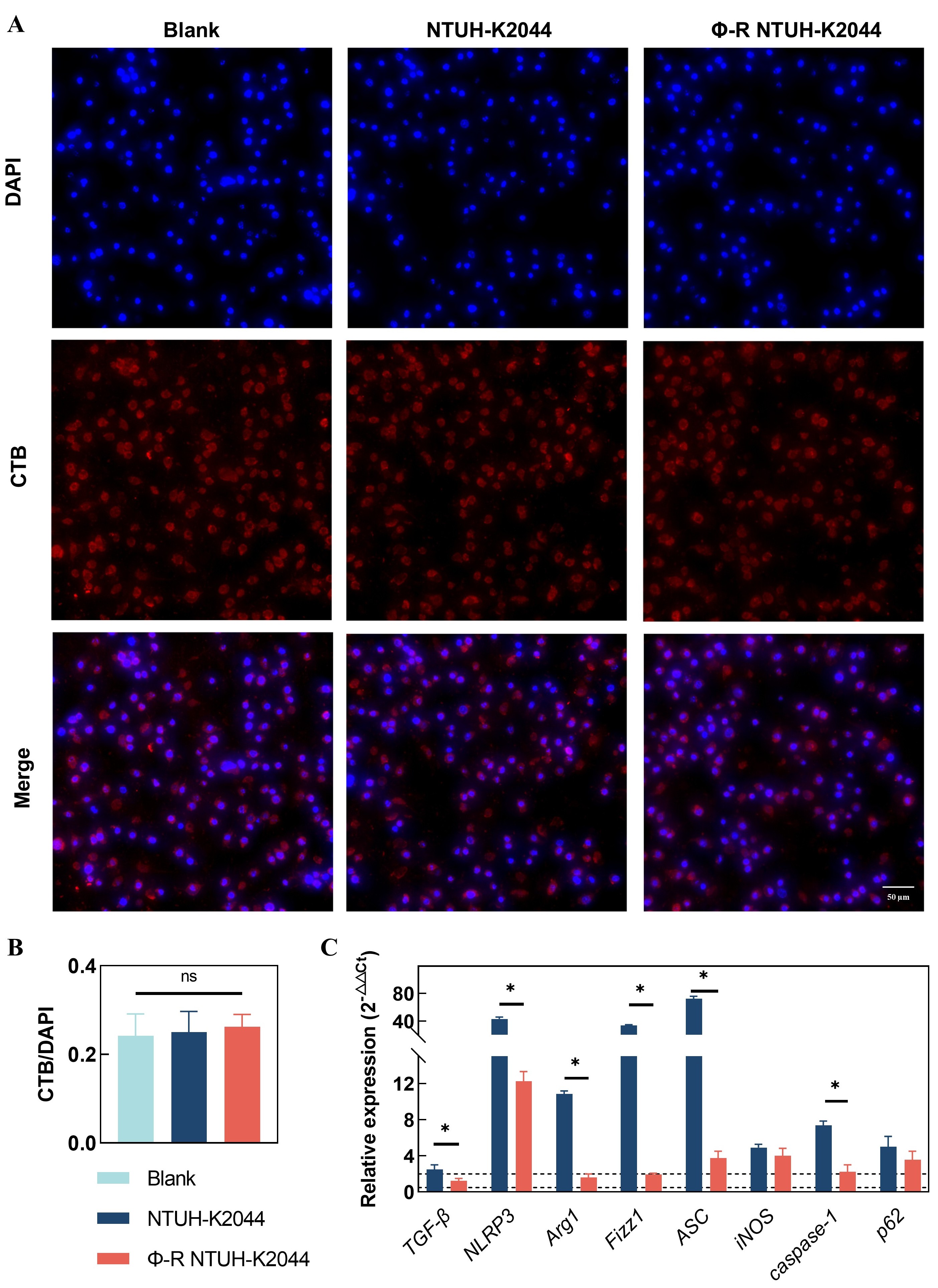

Supplement: S4 Fig.jpg [file KVIR_A_2600148_SM0640.jpg]
