# Supplementary material for: Evolving strategies of intracellular Hypervirulent Klebsiella pneumoniae during phage therapy: Reducing host autophagy and inflammation
Source: Virulence. 2025 Dec 4;16(1):2600148. doi: 10.1080/21505594.2025.2600148 (PMC12688233; doi:10.1080/21505594.2025.2600148)
Supplement: S3 Table_revised.docx [file KVIR_A_2600148_SM0647.docx]

S3 Table. Lung Tissue Injury Scoring System

|  | Score | | | | |
| --- | --- | --- | --- | --- | --- |
|  | 0 | 1 | 2 | 3 | 4 |
| Edema | none | <10% fields | 10–30% fields | 30–60% fields | >60% fields |
| Hemorrhage | none | ≤1/HPF | 2–3/HPF | >3/HPF or confluent foci; | Diffuse or multilobar |
| Inflammatory Cell Infiltration | none | scattered | small clusters (≤5 cells/HPF) | multiple clusters (>5 cells/HPF) | diffuse sheets |
| Small Airway Damage | none | <10% of small airways involved | 10–30% small airways involved | 30–60% small airways involved | >60% small airways involved |
| HPF ×400; 10 HPFs/section; 2–3 sections/mouse; scores defined as: edema %HPFs, hemorrhage foci/HPF, inflammation clusters/HPF, small-airway % obstruction (≈25%→3, 75%→4); total = 0–16. | | | | | |
