# Supplementary material for: Evolving strategies of intracellular Hypervirulent Klebsiella pneumoniae during phage therapy: Reducing host autophagy and inflammation
Source: Virulence. 2025 Dec 4;16(1):2600148. doi: 10.1080/21505594.2025.2600148 (PMC12688233; doi:10.1080/21505594.2025.2600148)
Supplement: S5 Table.docx [file KVIR_A_2600148_SM0645.docx]

S5 Table. qPCR primers for autophagy and inflammatory factor encoding genes

| Primer name | Sequence (5’ to 3’ direction) |
| --- | --- |
| *β-actin* | ATCTGGCACCACACCTTCTACAATG |
|  | CACGCTCGGTCAGGATCTTCATG |
| *NLRP3* | TCCGGCCTTACTTCAATCTG |
|  | TCACAGAGGAGCCTGAGTCC |
| *ASC* | TCAGAGTACAGCCAGAACAGG |
|  | CTCCAGGTCCATCACCAAGT |
| *caspase-1* | AGAGGATTTCTTAACGGATGCA |
|  | TCACAAGACCAGGCATATTCTT |
| *iNOS* | TGCCACGGACGAGACGGATAG |
|  | CTCTTCAAGCACCTCCAGGAACG |
| *p62* | AGGATGGGGACTTGGTTGC |
|  | TCACAGATCACATTGGGGTGC |
| *Fizz1* | TGACTGCTACTGGGTGTGCT |
|  | GGCAGTGGTCCAGTCAACG |
| *Arg1* | TGCTCACACTGACATCAACACTCC |
|  | TCTACGTCTCGCAAGCCAATGTAC |
| *TGF-β* | GCAACAATTCCTGGCGTTACCTTG |
|  | CAGCCACTGCCGTACAACTCC |
| *TNF-α* | GCGACGTGGAACTGGCAGAAG |
|  | GCCACAAGCAGGAATGAGAAGAGG |
| *IL-6* | ACTTCCATCCAGTTGCCTTCTTGG |
|  | TTAAGCCTCCGACTTGTGAAGTGG |
| *IL-1β* | TTGAAGAAGAGCCCATCCT |
|  | CGTTGCTTGGTTCTCCTTGT |
| *IL-18* | AGTAAGAGGACTGGCTGTGACC |
|  | TTGGCAAGCAAGAAAGTGTC |
| *IL-10* | CTGCTATGCTGCCTGCTCTTACTG |
|  | ATGTGGCTCTGGCCGACTGG |
| *soxS* | TATCAAAGATCGGCCGCTGG |
|  | AGTCGCCAGAAAGTCAGG |
| *oxyR* | TGCCGCTGTTCGATGAG |
|  | TTCAAAGCAGAAGCCCATCGC |
| *katG* | GATCCAGTTCGAAGCCAAAGACGC |
|  | CCTGCGGATCGTTGAGGAAG |
| *katE* | TGCTGAAATCAATGCCCGGGAC |
|  | GCTCGATCCCACCAAGC |
| *16S rRNA* | ATGACCAGCCACACTGGAAC |
|  | CTTCCTCCCCGCTGAAAGTA |
