# Supplementary material for: Evolving strategies of intracellular Hypervirulent Klebsiella pneumoniae during phage therapy: Reducing host autophagy and inflammation
Source: Virulence. 2025 Dec 4;16(1):2600148. doi: 10.1080/21505594.2025.2600148 (PMC12688233; doi:10.1080/21505594.2025.2600148)
Supplement: S1 Table.docx [file KVIR_A_2600148_SM0643.docx]

S1 Table. Scoring Criteria for Myocardial Injury Based on Histopathological Assessment

| Score | Criteria |
| --- | --- |
| 0 | Zero damage |
| 1 | (mild), interstitial edema and localized necrosis |
| 2 | (moderate), widespread myocardial cell swelling and necrosis |
| 3 | (severe), necrosis with contraction bands, neutrophil infiltration and compressed capillaries |
| 4 | (highly severe), diffuse necrosis with contraction bands, neutrophil infiltration, compressed capillaries and hemorrhage |
