# Supplementary material for: Evolving strategies of intracellular Hypervirulent Klebsiella pneumoniae during phage therapy: Reducing host autophagy and inflammation
Source: Virulence. 2025 Dec 4;16(1):2600148. doi: 10.1080/21505594.2025.2600148 (PMC12688233; doi:10.1080/21505594.2025.2600148)
Supplement: S2 Table.docx [file KVIR_A_2600148_SM0639.docx]

S2 Table. Modified Ishak Score for Liver Histology Activity Index

| Score | Ishak Grade |
| --- | --- |
| Periportal or periseptal interface hepatitis (piecemeal necrosis) | |
| 0 | None |
| 1 | Mild (focal, few portal areas) |
| 2 | Mild/moderate (focal, most portal areas) |
| 3 | Moderate (continuous around < 50% of tracts or septa) |
| 4 | Severe (continuous around > 50% of tracts or septa) |
| Confluent necrosis | |
| 0 | None |
| 1 | Focal confluent necrosis |
| 2 | Zone 3 necrosis in some areas |
| 3 | Zone 3 necrosis in most areas |
| 4 | Zone 3 necrosis + occasional portal–central bridging |
| 5 | Zone 3 necrosis + multiple portal–central bridging |
| 6 | Panacinar or multiacinar necrosis |
| Focal (spotty) lytic necrosis, apoptosis and focal inflammation | |
| 0 | None |
| 1 | One focus or less per 10× objective |
| 2 | Two to four foci per 10× objective |
| 3 | Five to ten foci per 10× objective |
| 4 | More than 10 foci per 10× objective |
| Portal inflammation | |
| 0 | None |
| 1 | Mild, some or all portal areas |
| 2 | Moderate, some or all portal areas |
| 3 | Moderate/marked, all portal areas |
| 4 | Marked, all portal areas |
| Ishak Stage | |
| 0 | No fibrosis |
| 1 | Fibrous expansion of some portal areas, with or without short fibrous septa |
| 2 | Fibrous expansion of most portal areas, with or without short fibrous septa |
| 3 | Fibrous expansion of most portal areas with occasional portal to portal bridging |
| 4 | Fibrous expansion of portal areas with marked bridging (portal to portal as well as portal to central) |
| 5 | Marked bridging (portal–portal and/or portal–central) with occasional nodules (incomplete cirrhosis) |
| 6 | Cirrhosis, probable or definite |
| The Ishak score separated the necroinflammatory components that are totaled to calculate the activity grade (0–18) from the stage (0–6), and varying degrees of confluent necrosis are listed in a separate necroinflammatory category. | |
